# A new approach to predict microhardness of two-phase in cutting S32760 duplex stainless steel

**DOI:** 10.1038/s41598-023-44708-0

**Published:** 2023-10-13

**Authors:** Xiangyuan Zhang, Lin Yang, Minli Zheng, Jialiang Liu, Mingjia Zhou, Fukang Gong

**Affiliations:** 1 Key Laboratory of Advanced Manufacturing and Intelligent Technology, Ministry of Education, Harbin University of Science and Technology, Harbin, 150080 People’s Republic of China; 2 College of Intelligent Manufacturing Engineering, Harbin Huade University, Harbin, People’s Republic of China

**Keywords:** Materials science, Mechanical engineering

## Abstract

The uneven distribution of microhardness in the two-phase structure of S32760 duplex stainless steel after cutting is attributed to variations in the crystal structure, which significantly impact the material's performance. This paper presents a new approach to predict the microhardness of two-phase based on the flow stresses in the austenitic and ferrite. The effect of strain, strain rate, and temperature on the flow stress in the shear plane of orthogonal cutting S32760 was analyzed, and the prediction model for microhardness of two-phase considering the two-phase flow stress was established to obtain a mapping relationship between the two-phase flow stress and the two-phase microhardness of S32760. The impact of cutting dosages on shear strain, strain rate, and temperature in the shear plane was investigated. A function relationship between cutting dosages and microhardness of austenite and ferrite in the shear plane was established, two-phase microhardness experiments were conducted, and the model's accuracy was validated with a prediction error of less than 6%. This study provided insights into the impact of strain, strain rate, and temperature in the shear plane on the microhardness of the two-phase, thus contributing to the theoretical foundation of processing techniques in duplex stainless steel.

## Introduction

Duplex stainless steel (DSS), known for its ferrite–austenite structure, finds extensive industrial use thanks to its commendable mechanical properties and corrosion resistance. It's worth noting that surface microhardness and corrosion resistance exhibit a close relationship^1^. Due to the differences in the crystal structure of the two-phase S32760, the microhardness of the two-phase is not uniformly distributed after cutting, which seriously affects the service performance of the material.

Bordinassi et al.^2^ discovered that austenite exhibits viscosity during deformation, a phenomenon attributed to grain rearrangement. This rearrangement in austenite is more time-dependent compared to the slip observed in ferrite. As a result, the deformation of austenite is highly influenced by the machining deformation rate. The cutting dosages that have the most significant effect on microhardness are the feed, and it is suggested that an increase in the tool-workpiece contact area and chip thickness, in addition to the thermal softening effect of increased feed rate, increases microhardness, cutting speed, and temperature. Krolczyk et al.^3^ investigated the surface microhardness gradient of duplex stainless steel during cutting. They observed that austenite exhibits stronger strain strengthening compared to ferrite, resulting in higher microhardness after cutting. Increasing the cutting speed during the turning process of duplex stainless steel can reduce the maximum microhardness by 10%. Apek et al.^4^, based on dislocation theory, propose that austenite is more prone to work hardening, while ferrite exhibits high strength. By measuring the surface integrity of the cut duplex stainless steel, it was found that due to the different yield ratios of the two-phase, austenite, as a plastic phase, undergoes more dislocation motion during deformation, resulting in higher residual stress. Ahmed et al.^5^ demonstrated the significant intensity of material deformation during the cutting of duplex stainless steel and also highlighted the substantial influence of tool geometry and cutting parameters on the resulting microstructure and properties, including the hardening of the machined surface. Based on previous research, it is evident that most studies begin with experimental phenomena. The process of microhardness testing and calculation necessitates meticulous microscopic observation and specialized equipment. Consequently, the research process often demands a substantial volume of experimental data for support. Mostly the objective of their investigation was the machined material or the tool. The interaction between the tool and the workpiece produces chips that play a crucial role in determining the nature of the machined surface and tool life^6^. While the microhardness of the shear plane can provide insights into the mechanical characteristics of the material within that plane^7^, there has been limited discussion on the microhardness changes in the chips generated during the cutting process. Additionally, there is a lack of analysis regarding the influence of cutting parameters (such as cutting depth and speed) on the microhardness of the two phases of chips. Furthermore, there is a need to investigate the mechanical behavior of austenite and ferrite when subjected to multiple physical fields during the cutting process.

This paper presents a predictive model for microhardness in a two-phase system, considering the flow stress of both austenite and ferrite. It also analyzes the effect of the cutting dosage of orthogonal cutting S32760 on the strain, strain rate, and temperature in the equipartition shear zone of the two-phase. Using the least squares regression model and prediction model for the microhardness of two-phase, establish a functional relationship between cutting dosages and the microhardness of austenite and ferrite in the shear plane. The variations in the two-phase microhardness of S32760 were analyzed during the cutting process under different physical fields. This analysis serves as a theoretical foundation for the optimization of the cutting techniques.

## Analysis of the relationship between two-phase flow stress and two-phase microhardness mapping for S32760

### Model of S32760 flow stress considering two-phase characteristics

Since S32760 consists of both austenite and ferrite, it is necessary to calculate the flow stresses of the two phases separately and then combine them using appropriate weighting for accumulation.

1 $$ \sigma = l_{1} \sigma_{1} + l_{2} \sigma_{2} $$

The constitutive equations for ferrite and austenite are denoted as $\sigma_{1} (\varepsilon ,\dot{\varepsilon },T)$ and $\sigma_{2} (\varepsilon ,\dot{\varepsilon },T)$, respectively. $\varepsilon$ represents the plastic strain, $\dot{\varepsilon }$ is the actual strain rate, and $T$ is the test temperature. Finally, $l_{1}$ and $l_{2}$ are the linear coefficients that indicate the proportion of ferrite and austenite in S32760.

The MTS (Mechanical Threshold Stress) model^8^ is a dynamic constitutive model that incorporates state variables to account for the influence of strain, strain rate, and temperature history on flow stress. It utilizes threshold stress as the sole internal variable to characterize the mechanical response of materials across an extremely broad range of strain rates. For both ferrite and austenite phases, their respective constitutive equations can be formulated as follows:

2 $$ \sigma_{1} = \sigma_{a1} + \sigma_{th1} = \sigma_{a1} + f(\dot{\varepsilon },T)\hat{\sigma }_{th1} $$

3 $$ \sigma_{2} = \sigma_{a2} + \sigma_{th2} = \sigma_{a2} + f(\dot{\varepsilon },T)\hat{\sigma }_{th2} $$

where $\sigma_{a1}$ and $\sigma_{a2}$ denote the non-thermal stress terms of ferrite and austenite, respectively, $f_{1} (\dot{\varepsilon },T)$ and $f_{2} (\dot{\varepsilon },T)$ represent two-phase thermal activation functions, respectively. In this context, $\widehat{\sigma }_{th1}$ and $\widehat{\sigma }_{th2}$ represent the thermal stress terms for ferrite and austenite, respectively.

In the case of the BCC structure, the thermal activation region remains unaffected by plastic strain, with the primary hindrance stemming from Peierls' internal stress^9^. The strain hardening term differs from the term that represents strain rate and temperature effects; instead, it is included in the non-thermal stress term. When considering FCC structures, it is crucial to account for the influence of material particle size on yield stress, and its precise form can be determined using the Hall–Petch relationship. The constitutive equations of ferrite and austenite include non-thermal stress terms specific to each phase, represented by Eqs. (4) and (5):

4 $$ \sigma_{a1} = m_{1} d_{1}^{{ - \frac{1}{2}}} + K_{1} \varepsilon^{{n_{1} }} + \sigma_{i1} $$

5 $$ \sigma_{{_{a2} }} = m_{{_{2} }} d_{{_{2} }}^{{ - \frac{1}{2}}} + \sigma_{{_{i2} }} $$

where $m_{1}$ and $m_{2}$ are constants that signify the strength of the grain boundaries in ferrite and austenite, while $d_{1}$ and $d_{2}$ denote the grain size of ferrite and austenite, respectively; $\varepsilon$ signifies the true strain, $K_{1}$ and $n_{1}$ represent the strain sensitivity index and strain hardening coefficient of the ferrite, respectively; $\sigma_{i1}$ and $\sigma_{i2}$ represent the stress induced by initial defects and impurities in ferrite and austenite, respectively.

The thermal activation function expression is^10^:

6 $$ f(\dot{\varepsilon },T) = \left\{ {1 - \left[ { - \frac{kT}{{G_{0} }}\ln (\frac{{\dot{\varepsilon }}}{{\dot{\varepsilon }_{0} }})} \right]^{\frac{1}{q}} } \right\}^{\frac{1}{p}} $$

Here, $T$ represents the temperature, $\dot{\varepsilon }_{0}$ denotes the reference strain rate, $G_{0}$ signifies the reference thermal activation energy, and $k$ represents Boltzmann’s constant. $p$ and $q$ are the barrier constants under consideration.

Since the BCC is not influenced by thermal stress and strain, the thermal stress component in ferrite is equivalent to its saturation stress value^10^. Nonetheless, given the high work hardening rate of austenite, it becomes imperative to account for the impact of strain rate and temperature on its strain hardening^11^. Thus, we can derive the following expression:

7 $$ \overset{\lower0.5em\hbox{$\smash{\scriptscriptstyle\frown}$}}{\sigma }_{th1} = \hat{\sigma }_{s1} e^{{\frac{kT}{{b^{3} Ea_{0} }}\ln \frac{{\dot{\varepsilon }}}{{\dot{\varepsilon }_{s0} }}}} $$

8 $$ \overset{\lower0.5em\hbox{$\smash{\scriptscriptstyle\frown}$}}{\sigma }_{th2} = K_{2} \varepsilon^{{n_{2} }} \hat{\sigma }_{s2} e^{{\frac{kT}{{b^{3} Ea_{0} }}\ln \frac{{\dot{\varepsilon }}}{{\dot{\varepsilon }_{s0} }}}} $$

where $\hat{\sigma }_{s1}$ represents the saturation threshold thermal component of ferrite at its reference value (T = 0 K), while $\hat{\sigma }_{s2}$ denotes the saturation threshold thermal component of ferrite, which is the threshold thermal stress at a strain hardening rate of 0; $b$ is the magnitude of the Burgers vector; $a_{0}$ is a constant coefficient, $E$ is the material shear modulus; $K_{2}$ and $n_{2}$ represent the strain sensitivity index and strain hardening coefficient of the austenite, respectively.

$\overline{\sigma } = l_{1} (\sigma_{i1} + m_{1} d_{1}^{{ - \frac{1}{2}}} ) + l_{2} (\sigma_{i2} + m_{2} d_{2}^{{ - \frac{1}{2}}} )$, $\overline{K} = l_{1} K_{1}$, $\hat{\sigma }_{th0} = l_{1} \hat{\sigma }_{s1}$, $\alpha = \frac{k}{{b^{3} Ea_{0} }}$, $\beta = \frac{k}{{G_{0} }}$, $\overline{Y} = l_{2} K_{2} \hat{\sigma }_{s2}$. The constitutive equation of S32760 can be expressed as follows

9 $$ \sigma = \overline{\sigma } + \overline{K}\varepsilon^{{n_{1} }} + (\widehat{Y}\varepsilon^{{n_{2} }} + \widehat{\sigma }_{th0} )\exp [\alpha T\ln (\frac{{\dot{\varepsilon }}}{{\dot{\varepsilon }_{s0} }})] \cdot \{ 1 - [ - \beta T\ln (\frac{{\dot{\varepsilon }}}{{\dot{\varepsilon }_{0} }})]^{\frac{1}{p}} \}^{\frac{1}{q}} $$

The constitutive parameters $\overline{\sigma }$, $\overline{K}$, and n1 are determined through experiments for the non-thermal stress term. Additionally, nine constitutive parameters (namely: $\widehat{Y}$, $\widehat{\sigma }_{th0}$, $n_{2}$, $\alpha$, $\beta$, $\dot{\varepsilon }_{s0}$, $\dot{\varepsilon }_{0}$, $p$, and $q$) are defined for the thermal stress term.

#### Non-thermal stress component of the flow stress

**Figure 1:**
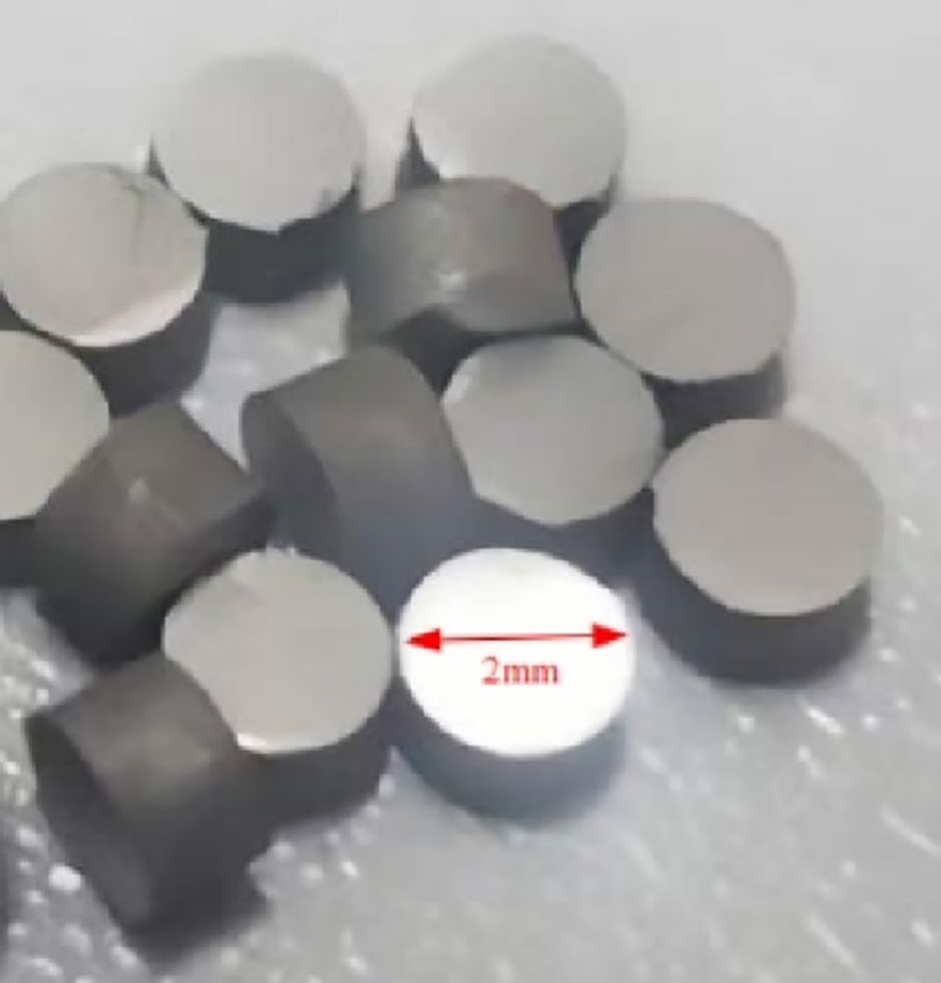
S32760 sample.

**Figure 2:**
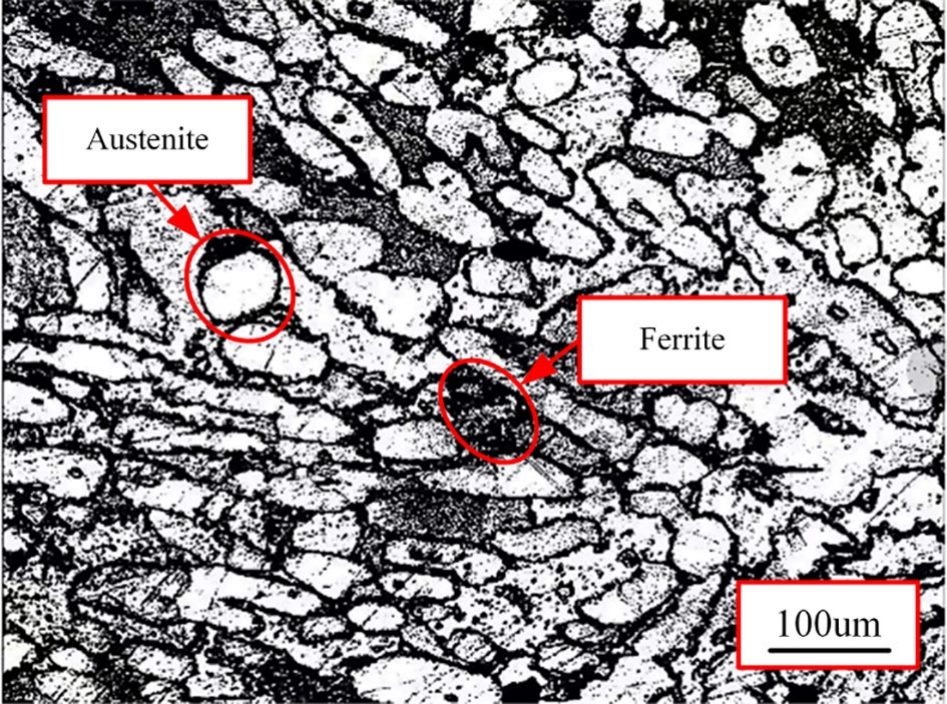
Microstructure of S32760.

The flow stress values at high strain rates are determined by fitting the S32760 two-phase constitutive equation. To achieve this, the stress–strain curve is evaluated through the Hopkinson pressure bar experiment. In our study, we used the ARCHIMEDES ALT1000 equipment for this purpose. The specimens used in the experiment were ϕ2 × 2 mm cylinders of S32760, as shown in Fig. 1. To minimize friction effects during the impact process, it was crucial to ensure that both ends of the sample were parallel and had the same surface roughness during sample preparation. Table 1 presents the key chemical constituents of S32760, while Table 2 provides the essential material parameters. Figure 2 shows the microstructure of S32760, observed through scanning electron microscopy (SEM).

**Table 1:** Chemical composition of S32760 (mass- %).

C	Cr	Cu	N	Mn	Ni	P	S	W	Mo	Si
0.018	25.5	0.75	0.2–0.3	0.53	7	0.017	0.001	0.75	3.5	0.42

**Table 2:** Mechanical and physical properties of S32760.

Melting temperature/°C	Density/kg/m^3^	Elastic modulus/GPa	Poisson’s ratio	Yield strength/MPa	Tensile strength/MPa	Microhardness of austenite/HV_0.05_	Microhardness of ferrite/HV_0.05_
1425	8000	204	0.3	596	964	317.5	351.3

**Figure 3:**
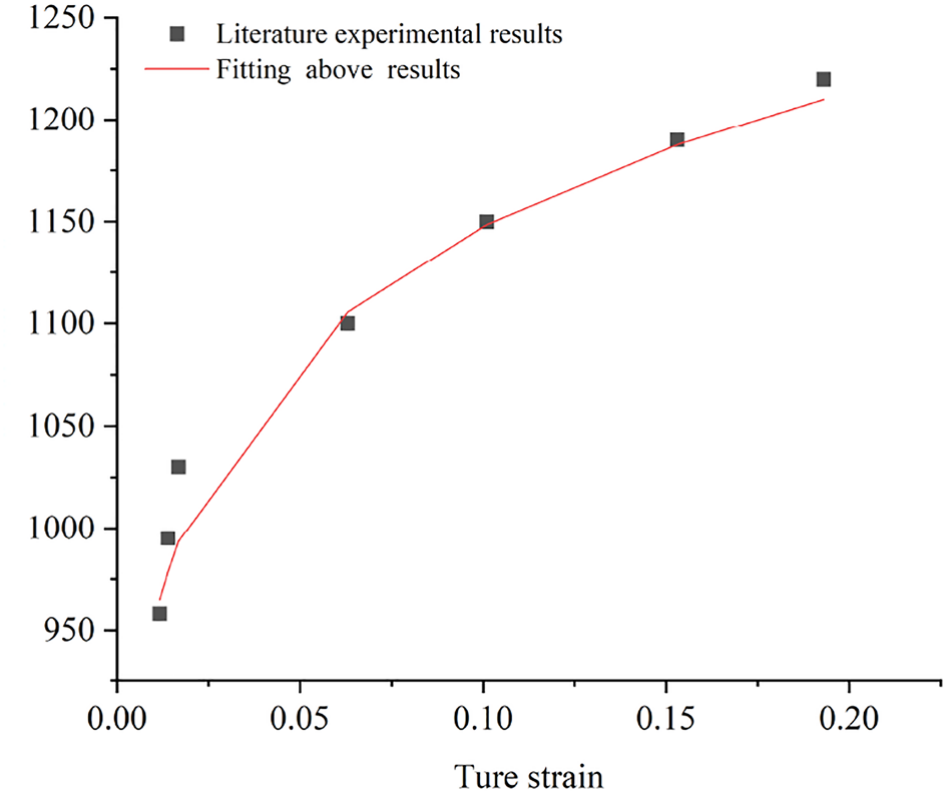
Fitting results of stress–strain curves at 500 °C and a strain rate of 10^4^ s^−1^.

As flow stress becomes temperature-independent at sufficiently high temperatures, we used the experimental data acquired at 500 °C and a strain rate of 104 s^−1^ to determine the non-thermal stress terms shown in Fig. 3. The non-thermal stress term yields three undetermined parameters: $\overline{\sigma } = 60$, $\overline{K} = 1318$, and $n_{1} = 0.07997$.

#### Thermal stress component of the flow stress

The thermal stress parameters $\widehat{Y}$, $\widehat{\sigma }_{th0}$, $n_{2}$, $\alpha$, $\beta$, $\dot{\varepsilon }_{s0}$, $\dot{\varepsilon }_{0}$, $p$, $q$ have been determined. However, it's important to highlight that parameters $\dot{\varepsilon }_{0}$ and $\dot{\varepsilon }_{s0}$, included within a logarithmic function, are not treated as separate, independent variables during the parameter fitting procedure. Their impact on the term is comparatively smaller than that of the previously mentioned parameters $\alpha$ and $\beta$. To accurately determine the parameters, predictions for these values are needed. Since S32760 consists of both BCC and FCC structures, its hexagonal close-packed (HCP) metal constitutive equation can be considered as a linear combination of FCC and BCC metal constitutive equations^12^. In this study, the reference strain rate of S32760 is determined by utilizing the reference strain rate of HCP metal.

The equation $\dot{\varepsilon }_{0} { = }\frac{{\dot{\gamma }_{0} }}{f(\gamma ,T)}$, as described by Nemat-Nasser et al.^13^, with $\dot{\gamma }_{0} = b\rho_{w} \omega_{0} l$, is employed with known values of $b = 0.28 \times 10^{ - 9}\, {\text{m}}$, $\omega_{0} = 10^{11}\, {\text{s}}^{ - 1}$, $l = 500{\text{b}}$, $\rho_{w} = 10^{15}\, {\text{m}}^{{ - 2}}$, $l_{0} = 500{\text{b}}$, at $T = 598\,{\text{K}}$ and $\gamma = 0.17$. After subsequent calculations, the value of $\dot{\varepsilon }_{0}$ is determined as $3 \times 10^{9} \,{\text{s}}^{{ - 1}}$. Given that ${\dot{\varepsilon }}_{s0}$ is typically two orders of magnitude greater than $\dot{\varepsilon }_{0}$, a value of $\dot{\varepsilon }_{s0} = 1 \times 10^{11} \,{\text{s}}^{{ - 1}}$ is considered appropriate^14^.

The remaining parameters can be determined through inverse identification, a process aimed at finding a set of material parameters that demonstrate a strong physical correlation. This correlation ensures a close match between the curve obtained from the constitutive equation and the experimental curve. The objective function is formulated based on the principles of the least squares method, with the aim of minimizing the sum of squared differences between the stress values calculated by the constitutive equation and the corresponding experimental measurements. The ultimate objective is to minimize this objective function. The expression of the objective function is given by:

10 $$ F(x) = \sum {_{i = 1}^{N} (Y_{i} (x) - Y_{i}^{*} )^{2} } $$

where $Y_{i}$ signifies the computed value at the ith point, $Y_{i}^{*}$ represents the experimental measurement obtained at the ith point, $x = [x_{1} ,x_{2} , \ldots ,x_{m} ]$ represents the parameter under optimization, and $N$ denotes the count of sampling points.

**Figure 4:**
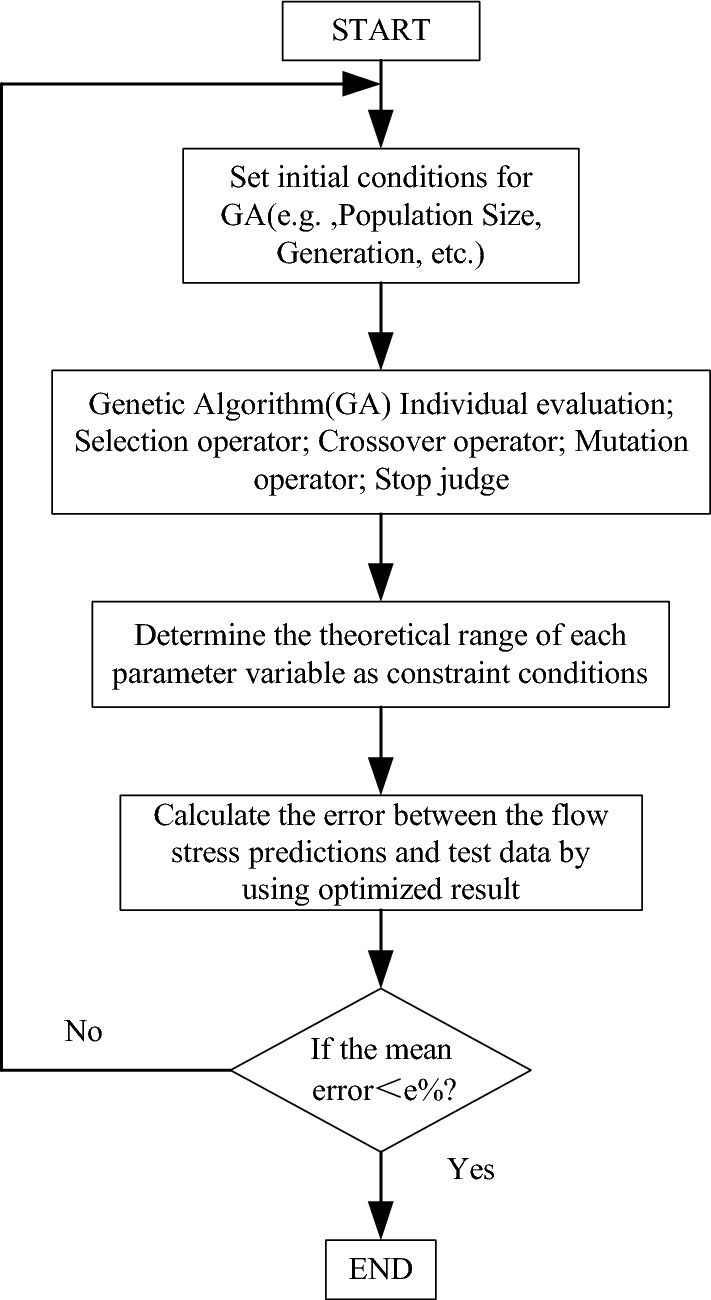
Constitutive model parameter optimization program flow chart.

The calculation of the objective function is optimized by utilizing the MATLAB program. Figure 4 presents the flowchart that illustrates the parameter optimization program for the constitutive model. This study proposes a combination of local algorithms and global genetic algorithms for parameter optimization. This approach not only enhances computational speed but also mitigates the undue reliance on initial values. It also alleviates the challenges associated with determining the global optimal solution^14^. Additionally, establishing the theoretical range for each parameter is crucial in ensuring accurate calculation results.

1. Determination of $\hat{Y}$ and $\hat{\sigma }_{th0}$: According to the research^13^, terms $1560 \times f(\gamma ,T)$ and $\hat{Y}\varepsilon^{{n_{2} }} + \hat{\sigma }_{th0}$ in Eq. (9) are categorized together, making them appropriate for establishing the ranges of $\hat{Y}$ and $\hat{\sigma }_{th0}$. The variable $\hat{\sigma }_{th0}$ is defined within the range of 1000 to 2000, with a central value at 1560, symmetrically extending in both directions. The temperature range, denoted as $T$, is defined from 77 to 998 K, while the strain range is constrained to values between 0 and 0.6. Consequently, the range of $\hat{Y}$ is estimated to be between 1500 and 3500.
2. Determination of $\alpha$ and $\beta$: The following values are associated with S32760 double stainless steel: $E = 159.2{\text{GPa}}$ and $a_{0} \in (0.2,2)$, Boltzmann’s constant $k = 1.38 \times 10^{ - 23} {\text{J/K}}$^15,16^, and the relationship $\alpha = k/(b^{3} Ea_{0} )$ is established. The parameter $\alpha \in (1.6 \times 10^{ - 6} ,1.6 \times 10^{ - 5} )$ is estimated and its range is expanded within the theoretically permissible maximum variation range. Given that parameters $\alpha$ and $\beta$ are within the same order of magnitude, it is reasonable to presume they share identical ranges.
3. Determination of $n_{2}$: The coefficient $n_{2}$ is widely accepted to be approximately 0.5 for most FCC metals, with minor variations among individual metals. For S32760, the theoretical range of maximum allowable variation can be considered (0,1].
4. Determination of $p$ and $q$: Correlation constants $p$ and $q$ determine the shape of the barrier. Typically, in the case of single crystals, the intervals $0 < p \le 1$ and $1 \le q < 2$ are employed. Examples of typical values, such as (2/3, 1), (1/2, 2), and (1, 2), correspond to rectangular, hyperbolic, and sinusoidal barriers, respectively. For most metals, ($p$, $q$) values such as (2/3, 1), (2/3, 2), (3/4, 4/3), and (1, 1) are considered, signifying a transition between rectangular and sinusoidal shapes. These values can also be used for S32760, as the $p$ and $q$ terms in the constitutive model average the same terms in the constitutive models of BCC and FCC structures.

The GA Optimization Toolbox is utilized to optimize the parameters using the genetic algorithm approach within the parameters range above, which yields a specific set of calculated constitutive model parameters for S32760. The determined parameters may serve as initial values for the constitutive parameters of S32760. The inverse identification process for the thermal stress term within the constitutive parameters is initiated using the orthogonal cutting experimental data as a reference point. The constitutive parameters undergo iterative adjustments until the simulated values closely align with the experimental data. These thermal stress parameters are presented in Table 3.

**Table 3:** Parameters in thermal stress component of S32760.

Undetermined constitutive parameters	The actual estimation range	Definite value	Unit
$\widehat{Y}$	[1500, 3500]	2148.0	MPa
$\hat{\sigma }_{th0}$	[1000, 2000]	1444.8	MPa
*n*_*2*_	(0, 1)	0.9985	/
$\alpha$	(10^−6^, 10^−4^)	9.384 × 10^−5^	1/K
$\beta$	(10^−6^, 10^−4^)	9.971 × 10^−5^	1/K
*p*	(0, 1]	0.99994	/
*q*	[1, 2)	1.00003	/

The developed S32760 constitutive model can be formulated as follows:

11 $$ \begin{aligned} & \sigma = 60 + 1318\varepsilon^{0.07997} + (2148\varepsilon^{0.9985} + 1444.4) \\ & \exp \left( {0.00009384T\ln \left( {\frac{{\dot{\varepsilon }}}{{10^{11} }}} \right)} \right)\left( {1 - \left( { - 0.00009971T\ln \left( {\frac{{\dot{\varepsilon }}}{{3 \times 10^{9} }}} \right)} \right)^{{\frac{1}{0.99994}}} } \right)^{{\frac{1}{1.00003}}} \\ \end{aligned} $$

### Equipartition shear zone model of orthogonal cutting based on Oxley theory

**Figure 5:**
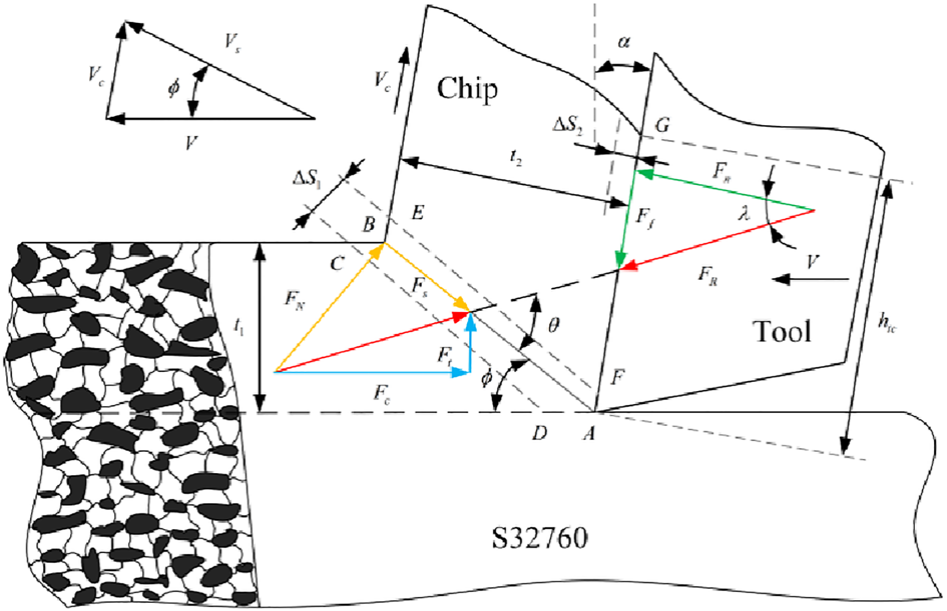
The parallel-sided shear plane of orthogonal cutting Diagram.

Oxley et al.^17^ initially presented the thick shear plane model, also known as the parallel-sided shear plane model, as a fundamental component of the predictive machining theory in Fig. 5. They developed an analysis framework that establishes a relationship between the process variables (material properties, tool geometry, cutting conditions) and output variables based on experimental observations of material deformation in the shear plane. The assumed conditions for this relationship are a steady cutting and plane strain. With the assistance of Oxley's predictive machining theory, it is now feasible to calculate theoretical values of output variables within the shear zone, including chip geometry, temperature, and cutting forces. Therefore, this study aims to develop a specialized prediction model specifically for S32760, focusing on orthogonal cutting forces.

#### Shear plane analysis

Within the diagram, $V$ represents the cutting speed, $V_{s}$ denotes the sliding speed of the chip along the shear plane direction, $V_{C}$ signifies the chip speed along the rake face direction, $\alpha$ represents the rake angle, and $\varphi$ represents the shear angle. By analyzing the vector relationship of velocity depicted in the figure, several inferences can be drawn:

12 $$ V_{s} = \frac{\cos \alpha }{{\cos (\phi - \alpha )}}V $$

13 $$ V_{c} = \frac{\sin \phi }{{\cos (\phi - \alpha )}}V $$

According to Oxley and Welsh^17^, the shear strain rate formula was derived through model simplification as follows:

14 $$ \dot{\gamma } = \frac{{V_{s} }}{{{\Delta }S_{1} }} $$

Here, $\Delta S_{1}$ is the thickness of the primary shear band. By substituting Eq. (13) into Eq. (14), we can obtain the shear strain rate using the following formula:

15 $$ \dot{\gamma } = \frac{\cos \alpha }{{{\Delta }_{s} \cos (\phi - \alpha )}}V $$

Considering that the AB plane resides in the middle of the primary shear band, the shear strain at AB is one-half of the shear strain observed in the main shear band, expressed as:

16 $$ \gamma_{AB} = \frac{1}{2}\frac{\cos \alpha }{{\sin \phi \cos (\phi - \alpha )}} $$

Based on the von Mises criterion, the equivalent strain and strain rate can be calculated as follows:

17 $$ \varepsilon_{AB} = \frac{{\gamma_{AB} }}{\sqrt 3 } = \frac{\cos \alpha }{{2\sqrt 3 \sin \phi \cos \left( {\phi - \alpha } \right)}} $$

18 $$ \dot{\varepsilon }_{AB} = \frac{{\dot{\gamma }}}{\sqrt 3 } = \frac{1}{\sqrt 3 } \cdot \frac{V\cos \alpha }{{\Delta S_{1} \cos (\varphi - \alpha )}} $$

In the chip formation model, $\lambda$ is the friction angle, and $\theta$ is the angle between $F_{R}$ and AB. The relationship mentioned above can be given by:

19 $$ \tan \theta = 1 + 2(\frac{\pi }{4} - \varphi ) - C_{0} $$

20 $$ \lambda = \theta + \alpha - \varphi $$

The modified strain rate constant $C_{0}$^18^ considers the impact of material strain and can be formulated as follows:

21 $$ C_{0} \approx C_{oxley} n\frac{{B\varepsilon_{AB}^{n} }}{{A + B\varepsilon_{AB}^{n} }} $$

where $C_{Oxley}$ denotes the ratio of the length of the shear plane ($l_{AB}$) to the thickness of the main shear band ($\Delta S_{1}$), A, B, and n are yield strength, strength coefficient, and strain hardening exponent in Johnson–Cook (J–C) constitutive parameters, respectively.

Cutting force $F_{c}$ and thrust force $F_{t}$ are components of the chip forming force $F_{R}$ that align parallel and perpendicular to the cutting direction. Furthermore, $F_{n}$ and $F_{f}$ denote the normal and frictional force at the tool-chip interface. Considering the relationship between the shear plane and the equilibrium conditions of the tool-chip contact region, we can deduce the following:

22 $$ F_{R} = \frac{{F_{s} }}{\cos \theta } = \frac{{K_{AB} t_{1} w}}{\sin \varphi \cos \theta } $$

23 $$ F_{c} = F_{R} \cos (\lambda - \alpha ) $$

24 $$ F_{t} = F_{R} \sin (\lambda - \alpha ) $$

25 $$ F_{n} = F_{R} \cos (\lambda ) $$

26 $$ F_{f} = F_{R} \sin \lambda $$

Iterative calculations are necessary to determine the average temperature due to the correlation between shear stress and temperature. The Boothroyd temperature model^19^ states the temperature increase in the primary shear band using the following equation:

27 $$ T_{AB} = T_{r} + \eta \Delta T_{SZ} $$

In this analysis,$T_{r}$ represents the initial temperature of the workpiece (set at 25 °C), $\eta$ indicates the percentage of total shear deformation energy converted into sensible heat (assumed to be 90%)^20^, and $\Delta T_{SZ}$ represents the temperature rise of the first deformation zone and can be expressed as follows:

28 $$ {\Delta }T_{SZ} = \frac{{(1 - \beta )F_{s} V_{s} }}{{m_{chip} C_{w} }} $$

Here, $C_{w}$ represents the specific heat of the workpiece; $F_{s} V_{s}$ stands for the work performed in the shear band, $m_{chip} = \rho Vt_{1} w$ signifies the chip mass per unit time, with $\rho$ representing the material density, $t_{1}$ denoting the undeformed chip thickness, and $w$ representing the cutting width. Additionally, $\beta$ accounts for the heat transferred from the shear plane to the workpiece, as described by the equation^18^:

29 $$ \beta = \left\{ {\begin{array}{*{20}c} {0.5 - 0.35\lg (R_{0} \tan \varphi )} & {0.04 \le R_{0} \tan \varphi \le 10} \\ {0.3 - 0.15\lg (R_{0} \tan \varphi )} & {R_{0} \tan \varphi > 10} \\ \end{array} } \right. $$

$R_{0}$ is the dimensionless heat value given by the following equation, while $K_{w}$ is the thermal conductivity of the workpiece material. $R_{0}$ is expressed as:

30 $$ R_{0} = \frac{{\rho C_{w} Vt_{1} }}{{K_{w} }} $$

The normal stress at point B can be determined by combining the stress boundary conditions at that point with the average value of shear stress in the shear zone using the following expression:

31 $$ \sigma^{\prime}_{N} = K_{AB} (1 + \frac{\pi }{2} - 2\alpha - 2C_{0} ) $$

The flow stress at the shear plane AB can be obtained by incorporating the two-phase properties into the flow stress model, yielding the following expression:

32 $$  K_{{AB}}  = \frac{1}{{\sqrt 3 }}\left\{ {\bar{\sigma } + \bar{K}\varepsilon _{{AB}} ^{{n_{1} }}  + \left( {\widehat{Y}\varepsilon _{{AB}} ^{{n_{2} }}  + \widehat{\sigma }_{{th0}} } \right)\exp \left[ {\alpha T_{{AB}} \ln \left( {\frac{{\dot{\varepsilon }_{{AB}} }}{{\dot{\varepsilon }_{{s0}} }}} \right)} \right] \cdot \left\{ {1 - [ - \beta T_{{AB}} \ln \left( {\frac{{\dot{\varepsilon }_{{AB}} }}{{\dot{\varepsilon }_{0} }}} \right)]^{{\frac{1}{p}}} } \right\}^{{\frac{1}{q}}} } \right\} $$

#### Tool-chip interface analysis

In the investigation of the secondary deformation zone, it is commonly assumed that the average thickness of the plastic deformation zone at the tool-chip interface can be represented by the equation $\Delta S_{2} = \delta t_{2}$. Here, $\delta$ represents the ratio between the thickness of the plastic zone at the tool-chip interface and the chip thickness. Consequently, the equivalent strain and equivalent strain rate at the tool-chip interface using the von Mises criterion is:

33 $$ \varepsilon_{{\text{int}}} = 2\varepsilon_{AB} + \frac{1}{\sqrt 3 } \cdot \frac{{h_{tc} }}{{\delta t_{2} }} $$

34 $$ \dot{\varepsilon }_{{\text{int}}} = \frac{1}{\sqrt 3 } \cdot \frac{{V_{c} }}{{\delta t_{2} }} $$

The tool-chip interface length $h_{tc}$ can be determined by:

35 $$ h_{tc} = \frac{{t_{1} \sin \theta }}{\cos \lambda \sin \varphi }(1 + \frac{{C_{0} }}{3\tan \theta }) $$

Assuming a uniform distribution of normal stress at the tool-chip interface, we can express the equations for the tool-chip interface stress $\tau_{{\text{int}}}$ and the normal stress $\sigma_{N}$ at point B as:

36 $$ \tau_{{\text{int}}} = \frac{{F_{f} }}{{h_{tc} w}} $$

37 $$ \sigma_{N} = \frac{{F_{N} }}{{h_{tc} w}} $$

The average temperature $T_{{\text{int}}}$ at the tool-chip interface is given by:

38 $$ T_{{\text{int}}} = T_{r} + \Delta T_{sz} + \Psi \Delta T_{M} $$

where $\Delta T_{M}$ is the maximum temperature rise in chips; $\psi$ is the partition coefficient of $\Delta T_{M}$ to the tool-chip interface ($\psi = 0.6$ in this analysis)^21^.

By considering a rectangular heat source at the interface, the equation developed by Boothroyd^19^ is as follows:

39 $$ \lg (\frac{{\Delta T_{M} }}{{\Delta T_{C} }}) = 0.06 - \delta \sqrt {\frac{{R_{0} t_{2} }}{{t_{1} }}} + 0.5\lg (\frac{{R_{0} t_{2} }}{{h_{tc} }}) $$

40 $$ \Delta T_{c} = \frac{{F_{f} V_{c} }}{{m_{chip} C_{w} }} $$

where $\Delta T_{C}$ is the average temperature rise in chip.

The flow stress at the tool-chip interface can be obtained by incorporating the two-phase properties into the flow stress model, yielding the following expression:

41 $$  K_{{chip}}  = \frac{1}{{\sqrt 3 }}\left\{ {\bar{\sigma } + \bar{K}\varepsilon _{{\text{int} }}^{{n_{1} }}  + \left( {\widehat{Y}\varepsilon _{{\text{int} }} ^{{n_{2} }}  + \widehat{\sigma }_{{th0}} } \right)\exp \left[ {\alpha T_{{\text{int} }} \ln \left( {\frac{{\dot{\varepsilon }_{{\text{int} }} }}{{\dot{\varepsilon }_{{s0}} }}} \right)} \right] \cdot \left\{ {1 - \left[ { - \beta T_{{\text{int} }} \ln \left( {\frac{{\dot{\varepsilon }_{{\text{int} }} }}{{\dot{\varepsilon }_{0} }}} \right)} \right]^{{\frac{1}{p}}} } \right\}^{{\frac{1}{q}}} } \right\} $$

#### Modeling chip formation forces

**Figure 6:**
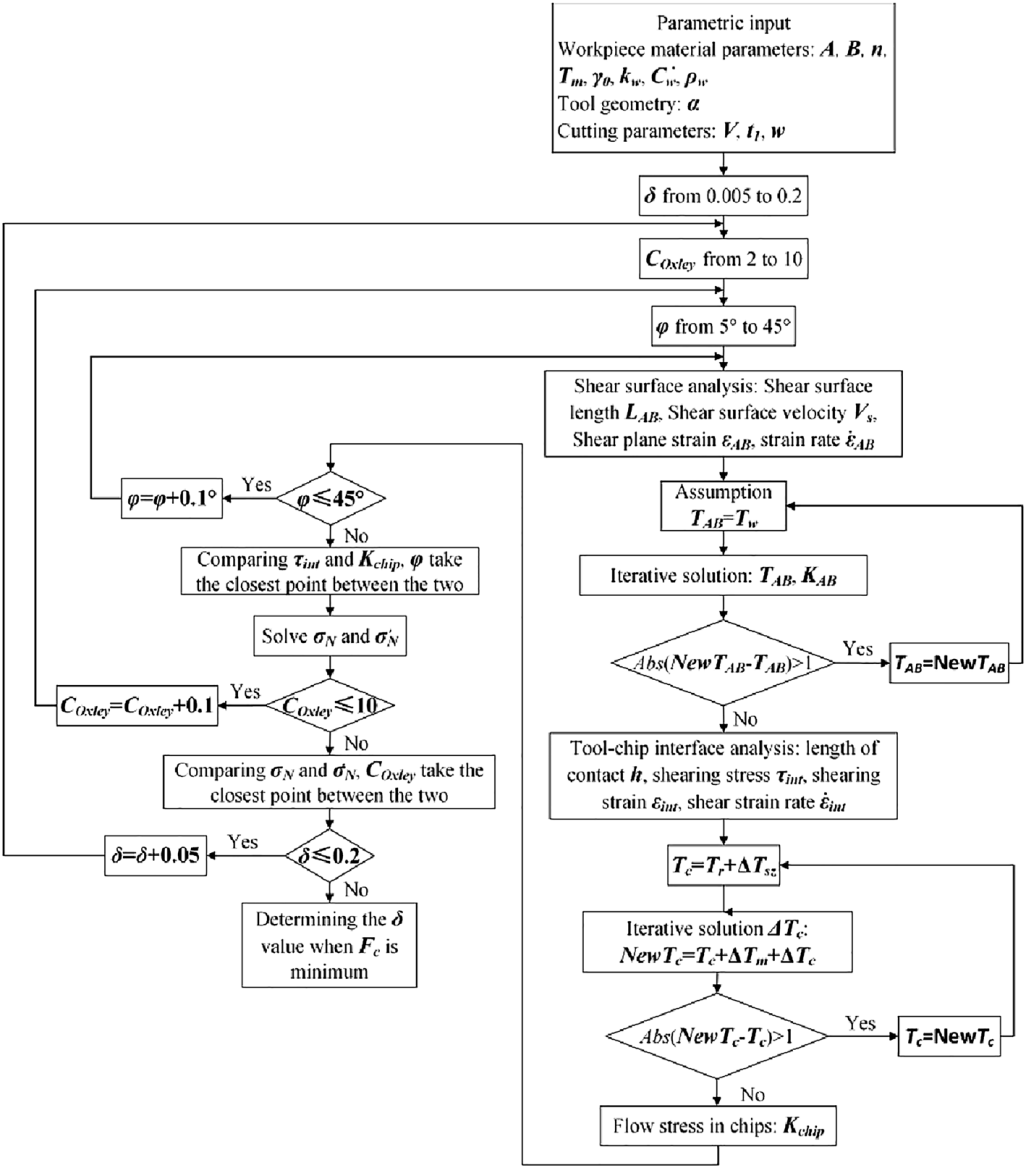
Calculation flow chart of the orthogonal cutting process.

The computational procedure for the orthogonal cutting process, accounting for variations in cutting conditions, material properties, and tool geometry, is illustrated in Fig. 6.

The deformation coefficient of the shear zone ($C_{Oxley}$), the shear angle ($\varphi$), and the ratio of secondary deformation zone thickness to chip thickness ($\delta$) are investigated iteratively within the specified ranges: $C_{Oxley} \in [2,10][2,10]$, $\varphi \in [5^\circ ,45^\circ ]$, and $\delta \in [0.005,0.2]$^19^. Conformity with three equilibrium conditions signifies the conclusion of the calculation process, following Oxley's cutting theory. These conditions involve (1) Stress equilibrium at the tool-chip interface, represented by $\sigma_{chip} = \tau_{int}$; (2) Stress equilibrium at the tooltip-chip interface, described as $\sigma^{\prime}_{N} = \sigma_{N}$; and (3) The principle of minimizing cutting force ($F_{c}$)^22^.

The temperature of the shear surface in the cutting force prediction model is calculated by determining the flow stress of the initial temperature and updating it based on the flow stress. This iteration process continues until the temperature difference is below 1 °C. Similarly, the initial temperature for the tool-chip interface includes the temperature rise from room temperature and the first deformation zone. The chip temperature is updated incrementally until the temperature difference is below 1 °C. The values of $\tau_{{\text{int}}}$ and $K_{chip}$ are compared, and the $\varphi$ value with the smallest discrepancy between $\tau_{{\text{int}}}$ and $K_{chip}$ is selected. Subsequently, this shear angle is applied to calculate $\sigma_{N}$ and $\sigma^{\prime}_{N}$. Likewise, the comparison between $\sigma_{N}$ and $\sigma^{\prime}_{N}$ is continued, and the ‘$C_{Oxley}$’ value at the point of minimal difference is chosen. Further parameters of the shear zone are then calculated, and the value of $\delta$ is determined based on the minimum shear force, denoted as $F_{c}$.

### Model validation

**Figure 7:**
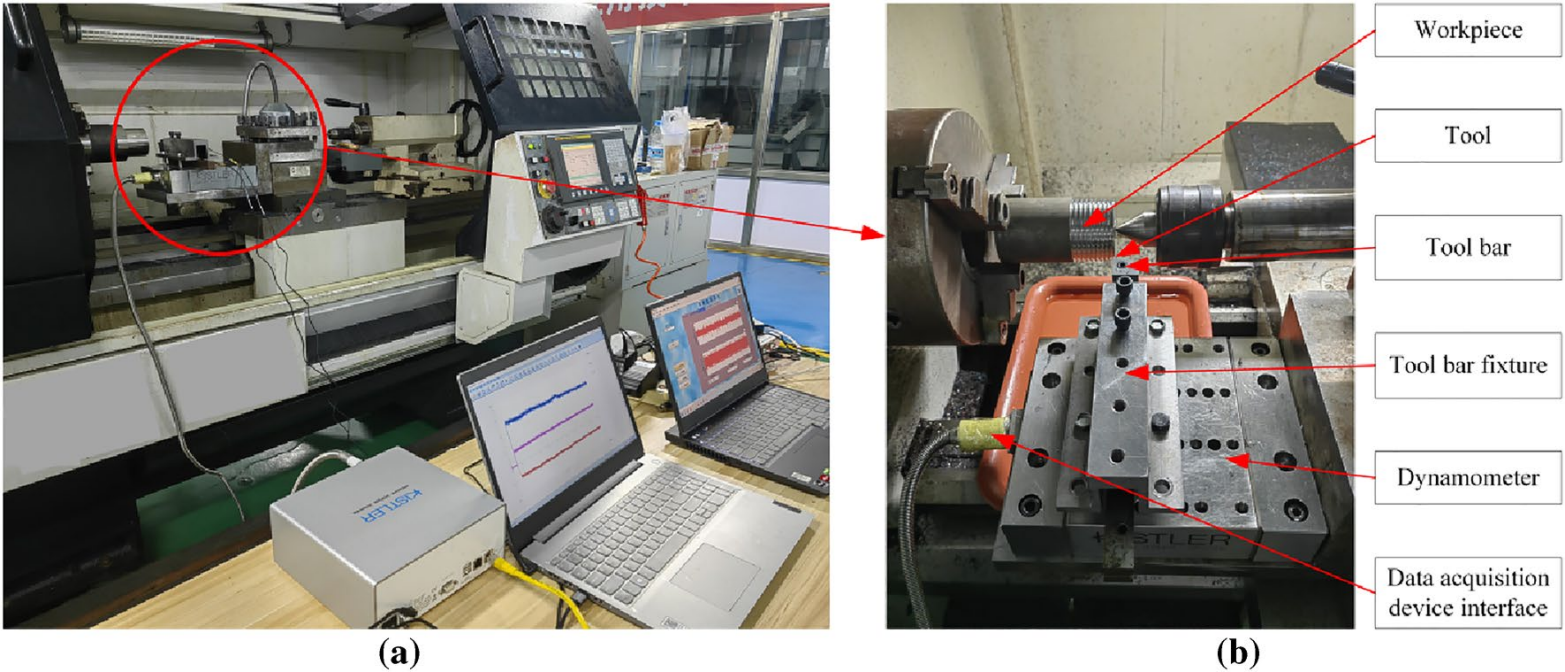
Orthogonal cutting test platform. (**a**) Global view; (**b**) detail view.

**Figure 8:**
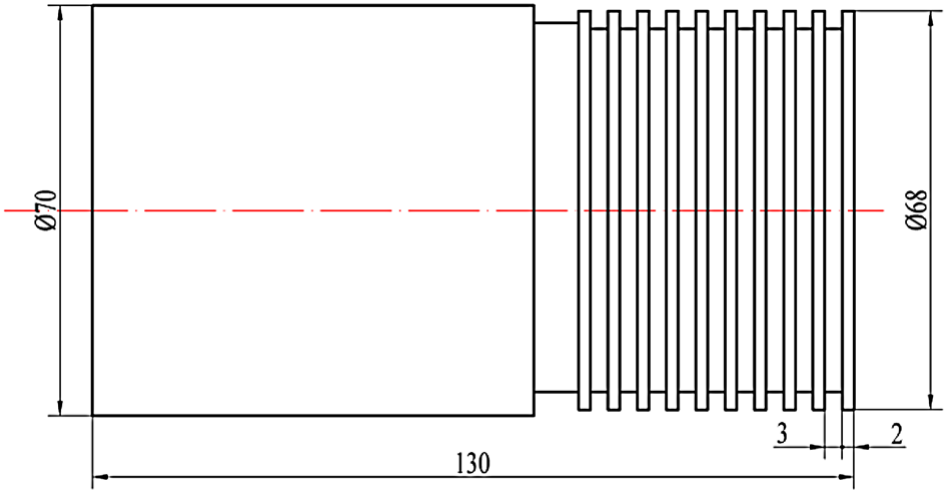
Diagram of the orthogonal cutting sample.

A test platform for orthogonal cutting of S32760 was constructed. The platform consists of a ϕ70 mm × 130 mm bar stock securely clamped using a three-jaw fixture, as shown in Fig. 7. Rough turning was conducted with a cutting depth of 1 mm, followed by grooving with a width of 3 mm. Multiple grooves, spaced 2 mm apart, were created at a depth of 3 mm, as demonstrated in Fig. 8. The tool employed was the MGGN300-V DH8532 model, with rake and back angles set at 18° and 8°, respectively, and a blade inclination angle of 0°. The tool holder utilized was the MGEHR2525-3 model. Turning and grooving operations were conducted on a CK6150 lathe manufactured by Dalian Machine Tool Works. The cutting tool employed is the MGGN300-V DH8532 model, with a rake angle set at 18°, a caster angle set at 8°, and a blade tilt angle of 0°. To guarantee accuracy, every experiment was methodically repeated three times, followed by the computation of the mean value. The cutting force prediction model was validated using the orthogonal cutting conditions presented in Table 4.

**Table 4:** Orthogonal cutting conditions.

Experimental group number	Cutting speed m/min	Feed rate mm/r	Cutting depth mm	Experimental group number	Cutting speed m/min	Feed rate mm/r	Cutting depth mm
1	63	0.3	3	9	63	0.5	3
2	106	0.3		10	106	0.5	
3	148	0.3		11	148	0.5	
4	214	0.3		12	214	0.5	
5	63	0.4		13	63	0.6	
6	106	0.4		14	106	0.6	
7	148	0.4		15	148	0.6	
8	214	0.4		16	214	0.6	

**Figure 9:**
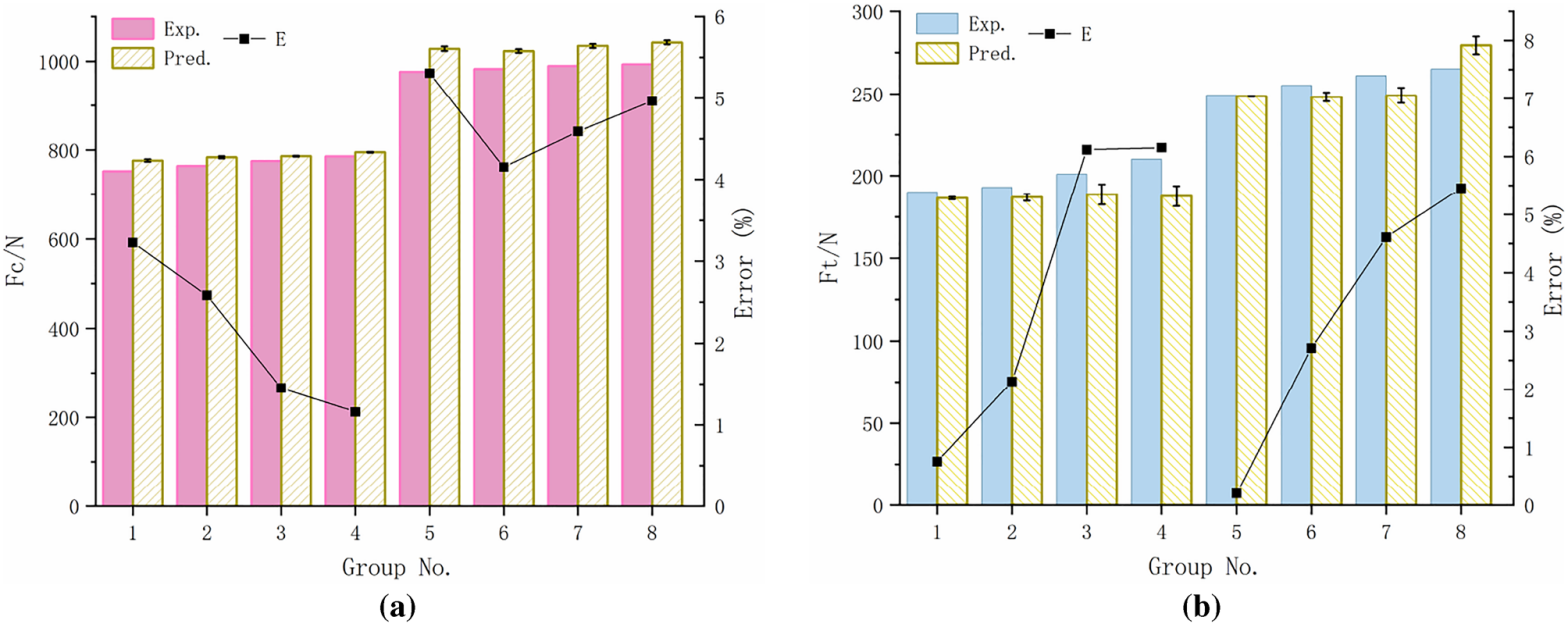
Comparison of predicted and measured cutting forces: (**a**) $F_{c}$; (**b**) $F_{t}$.

In the orthogonal cutting experiments, the measured cutting forces of eight groups with feed rates of 0.3 mm/r and 0.4 mm/r were compared with the predicted cutting forces, as illustrated in Fig. 9. The equipartition shear zone model for orthogonal cutting enhances the precision of cutting force predictions.

### Microhardness prediction model considering two-phase flow stresses

During the cutting process, the workpiece material in the shear plane experiences various effects, such as strain hardening, strain rate reinforcement, and thermal softening, which arise due to the force-thermal coupling^23–25^. Sonmez and Demir derived an analytical relationship between hardness and flow stress using the Brinell and Vickers methods. The hardness can be approximated by the following expression^26^:

42 $$ H = C\sigma $$

where $C$ is a constant, which is approximately equal to 3, $H$ is the microhardness, and $\sigma$ is the flow stress.

Following the equipartition shear zone model, the two-phase flow stress can be estimated as $\sqrt 3$ times the shear stress. Additionally, the microhardness of ferrite and austenite has been defined using the formula 1 HV = 1 kg *f* mm^−2^ = 9.8 N mm^−2^ = 9.8 Mpa. The microhardness values for ferrite and austenite have been expressed as

43 $$ HV_{1} = \frac{3}{9.8}\sqrt 3 \sigma_{1} $$

44 $$ HV_{2} = \frac{3}{9.8}\sqrt 3 \sigma_{2} $$

where $HV_{1}$ and $HV_{2}$ are the microhardness of ferrite and austenite, respectively; $\sigma_{1}$ and $\sigma_{2}$ are the shear stress of ferrite and austenite, respectively.

## Analysis of the effect of cutting dosage on the multi-physical field in the shear plane

According to the first part of "Equipartition shear zone model of orthogonal cutting based on Oxley theory" section, the shear angle $\varphi$, shear plane temperature $T_{AB}$, shear plane strain $\varepsilon_{AB}$, shear plane strain rate $\dot{\varepsilon }_{AB}$, and shear stress $K_{AB}$ were extracted according to the orthogonal cutting equipartition shear zone model, as shown in Table 5.

**Table 5:** Multi-physics field distribution in the shear plane at different cutting dosages.

Experimental group number	$\varphi /^\circ$	*T*_*AB*_/K	*ε*_*AB*_	$\dot{\varepsilon }_{AB} /{\text{s}}^{ - 1}$	*K*_*AB*_/Mpa
1	40.56	752.35	0.4456	10,416	770.46
2	40.52	772.77	0.4458	17,509	776.68
3	40.50	786.26	0.4459	24,432	781.01
4	40.47	801.58	0.4461	35,300	786.1
5	40.62	753.21	0.4452	7825.4	760.76
6	40.59	774.16	0.4454	13,156	766.26
7	40.57	787.41	0.4455	18,359	770.41
8	40.54	802.46	0.4457	26,524	775.29
9	40.65	754.85	0.445	6166.1	753.02
10	40.63	775.41	0.4451	10,536	758.41
11	40.60	788.62	0.4453	14,698	762.35
12	40.56	803.61	0.4456	21,229	766.99
13	40.69	756.07	0.4447	5227.4	746.97
14	40.66	775.54	0.445	8787.2	752.65
15	40.63	789.57	0.4452	12,258	756
16	40.61	804.31	0.4453	17,716	760.65

During the cutting process, chips experience deformation and thermal loads, leading to intricate changes in the microhardness of the two-phase material in the shear plane. These changes arise from the combined effects of multiple physical fields. Based on the analysis model of the orthogonal cutting equipartition shear zone with two-phase flow stress characteristics, a mapping relationship between the multi-physics fields and the cutting dosages is established.

**Figure 10:**
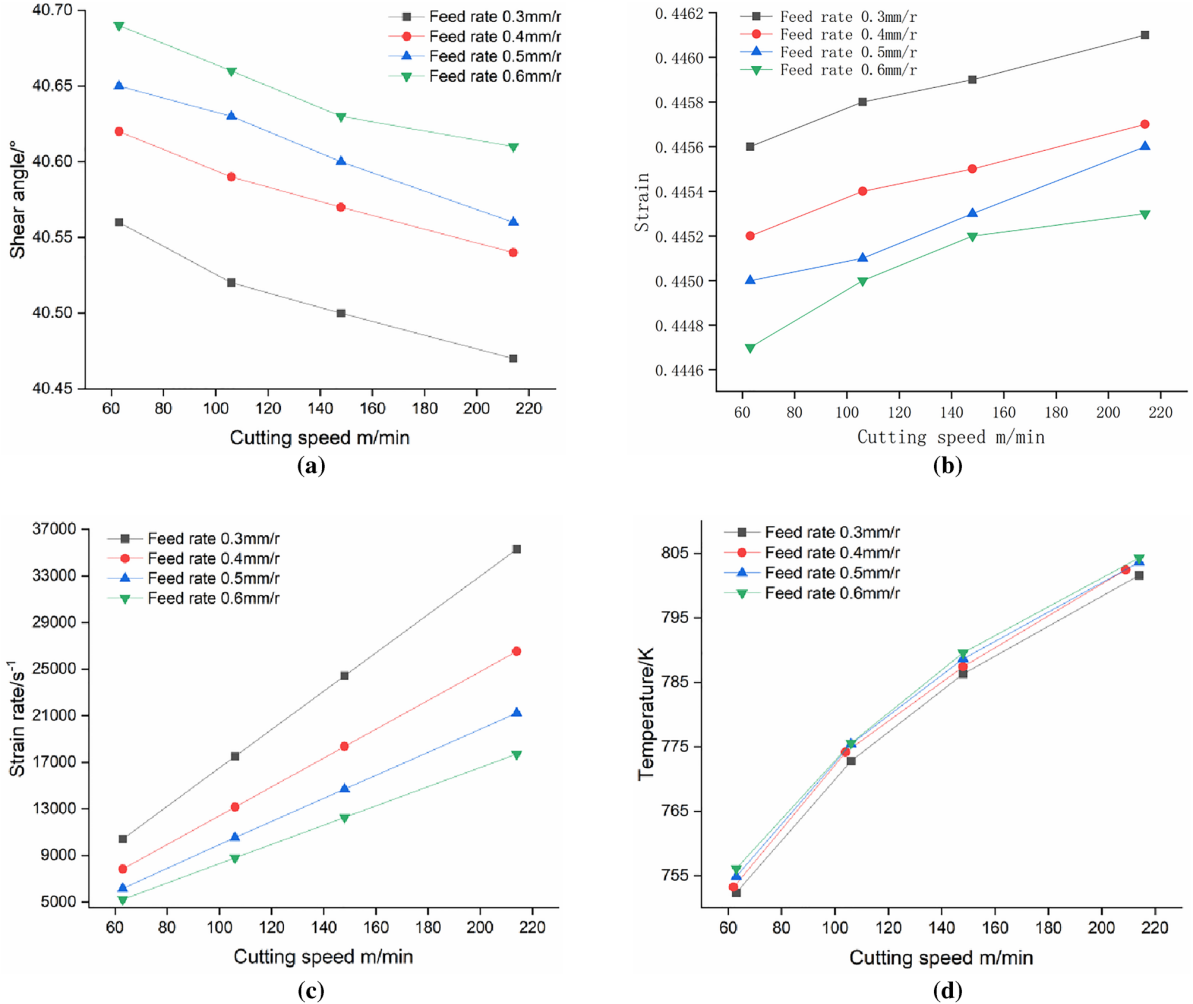
The effect of cutting dosages on shear parameters: (**a**) shear angle; (**b**) strain; (**c**) strain rate; and (**d**) temperature.

Figure 10 presents the analysis of the impact of cutting parameters on shear angle, strain, strain rate, and temperature by comparing experimental data from groups 1–16. In Fig. 10a, it can be observed that the shear angle decreases as the cutting speed increases, whereas it increases with an increase in feed rate. This phenomenon can be attributed to the enhanced plastic deformation of the workpiece material at the shear plane caused by higher cutting speeds. However, increased feed rates reduce material plastic deformation and limit chip shear slip distance. The analysis in Fig. 10b reveals a gradual increase in strain within the shear zone as cutting speed increases, whereas an increase in feed rate leads to a decrease in strain. In Fig. 10c, a nearly linear correlation is evident between the shear strain rate and cutting speed, with higher feed rates associated with a decrease in the shear strain rate. Figure 10d presents an increasing trend in average temperature within the shear plane as cutting speed increases. However, the influence of increasing feed on temperature is relatively insignificant. The decrease in shear strain rate as feed rates increase can be attributed to the greater cutting forces and heat generation. However, the longer working length of the cutting edge results in a larger surface area available for heat dissipation. As a result, despite the increased cutting heat, the temperature rise is constrained.

## Regression prediction model for two-phase microhardness

### Predicted microhardness of two-phase

The average shear stress within the shear plane is determined by applying the two-phase stress mixing rule. Utilizing a universally applicable method proposed for analyzing multiphase materials^27^, the shear strain of both phases at the shear plane can be calculated using Eqs. (45) and (46):

45 $$ \sigma (\varepsilon ) = F \cdot \sigma_{1} (\varepsilon_{1} ) + (1 - F) \cdot \sigma_{2} (\varepsilon_{2} ) $$

46 $$ \varepsilon = F \cdot \varepsilon_{1} + (1 - F) \cdot \varepsilon_{2} $$

where $\varepsilon_{1}$ and $\varepsilon_{2}$ are the strain values for the ferrite phase and the austenitic phases, respectively.

The S32760 matrix was observed using scanning electron microscopy (SEM), and the proportion of the two phases was analyzed using Image J software. Based on the analysis conducted with Image J software, it was determined that the volume fraction ratio of ferrite to austenite was 27/23, resulting in an F value of 0.54.

By substituting the shear strain, average shear strain rate, average temperature, and shear stress of austenite and ferrite in Table 5 into Eqs. (45) and (46), we can determine the respective strain and stress for austenite and ferrite. Based on these results, the microhardness of the two phases can be calculated using Eqs. (43) and (44). The corresponding values are tabulated in Table 6.

**Table 6:** Shear strain, stress, and microhardness of two-phase in the shear plane.

Experimental group number	Shear strain of ferrite	Shear strain of austenite	Shear stress of ferrite /Mpa	Shear stress of austenite /Mpa	Predicted microhardness of ferrite/HV	Predicted microhardness of austenite /HV
1	0.4457	0.4455	770.47	770.44	408.52	408.5
2	0.4463	0.4452	776.75	776.58	411.85	411.76
3	0.4458	0.446	781.12	781.14	414.16	414.18
4	0.4638	0.4253	788.9	782.67	418.29	414.99
5	0.4283	0.4651	758.37	763.49	402.1	404.81
6	0.3808	0.5212	756.35	776.49	401.03	411.71
7	0.4459	0.4449	770.47	770.32	408.52	408.44
8	0.4849	0.3997	781.04	768.01	414.12	407.21
9	0.4454	0.4446	752.59	752.49	399.04	398.98
10	0.452	0.437	759.34	757.28	402.62	401.52
11	0.4453	0.4453	762.35	762.34	404.21	404.21
12	0.4456	0.4455	767	766.98	406.68	406.67
13	0.4447	0.4447	746.96	746.96	396.05	396.05
14	0.4117	0.4840	748.09	757.62	396.65	401.71
15	0.4675	0.4191	758.96	752.38	402.41	398.93
16	0.4454	0.4452	760.57	760.55	403.27	403.26

### Microhardness prediction model revision

Based on the two microhardness predictions, a quadratic polynomial regression model was selected to establish a mathematical model between the cutting speed $v_{c}$ and the feed rate $f$ on the microhardness of the two-phase of the chip shear plane, using the principle of least squares, as follows:

47 $$ HV_{1} = a_{0} + a_{1} v_{c} + a_{2} f + a_{s} v_{c}^{2} + a_{4} f^{2} + a_{5} v_{c} f $$

48 $$ HV_{2} = b_{0} + b_{1} v_{c} + b_{2} f + b_{s} v_{c}^{2} + b_{4} f^{2} + b_{5} v_{c} f $$

where $a_{0} \sim a_{5}$ are the ferrite variable parameters, and $b_{0} \sim b_{5}$ are the austenite variable parameters.

Using Matlab software to fit a multiple regression, the least squares regression equations for the microhardness of the ferrite and austenite with respect to cutting speed $v_{c}$ and feed rate $f$ were obtained as follows:

49 $$ HV_{1} = 427.9271 + 0.0983v_{c} - 107.2943f + 80.5f^{2} - 0.07v_{c} f $$

50 $$ HV_{2} = 423.7528 + 0.1126v_{c} - 79.6526f - 0.0003v_{c}^{2} + 36.4375f^{2} + 0.0343v_{c} f $$

The significance analysis of the regression equations is shown in Table 7. The established regression models have $P$ values that are all less than 0.005, indicating their high significance. The determination coefficient ($R^{2}$) values from the regression models demonstrate their capacity to explain 90.66% and 94.78% of the microhardness variability in ferrite and austenite, respectively, within the chip shear band. The coefficient of variation ($CV$) values with the regression models for the microhardness of ferrite and austenite are 0.41% and 0.36%, respectively, indicating their high reliability. Therefore, the established regression equations can be used to predict the microhardness of two-phase in the chip shear band within a reasonable range of cutting dosages.

**Table 7:** Significance analysis of the regression equations for microhardness of ferrite and austenite.

	P-value	F-value	R^2^-value	CV-value
*HV*_*1*_	0.0045	9.7028	0.9066	0.0041
*HV*_*2*_	4.6965e-04	18.1534	0.9478	0.0036

### Experimental validation of the two-phase microhardness prediction model

**Figure 11:**
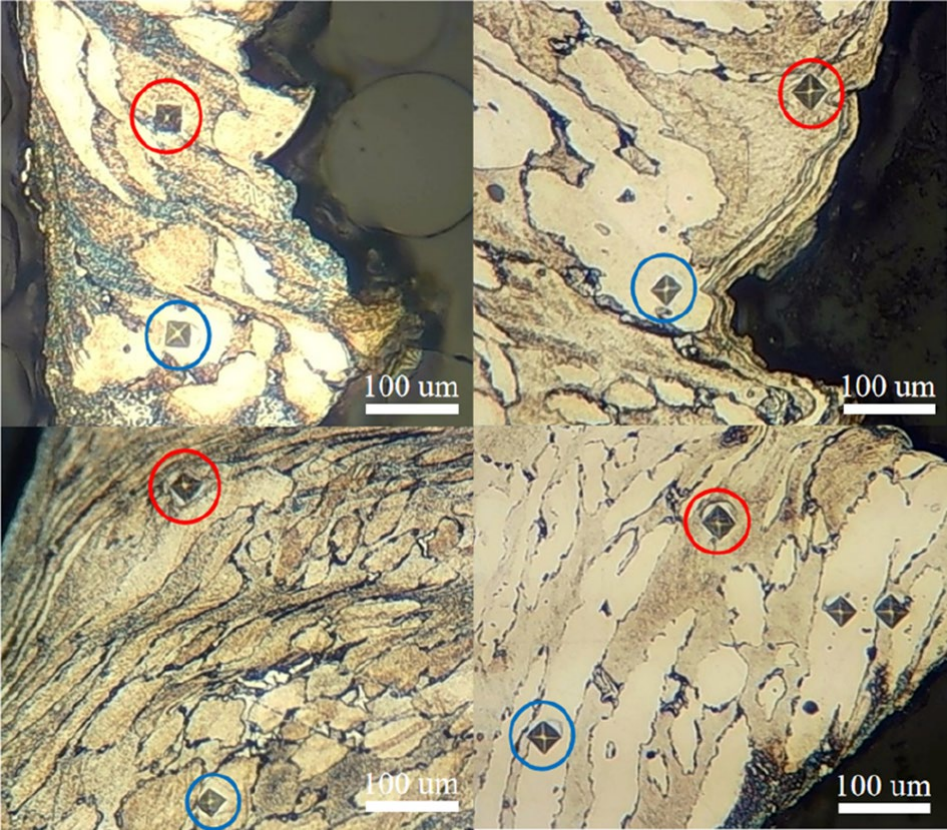
Indentation metallographs of the two-phase microhardness of the chips in the experiment, where the blue circle represents ferrite and the red circle represents austenite.

**Figure 12:**
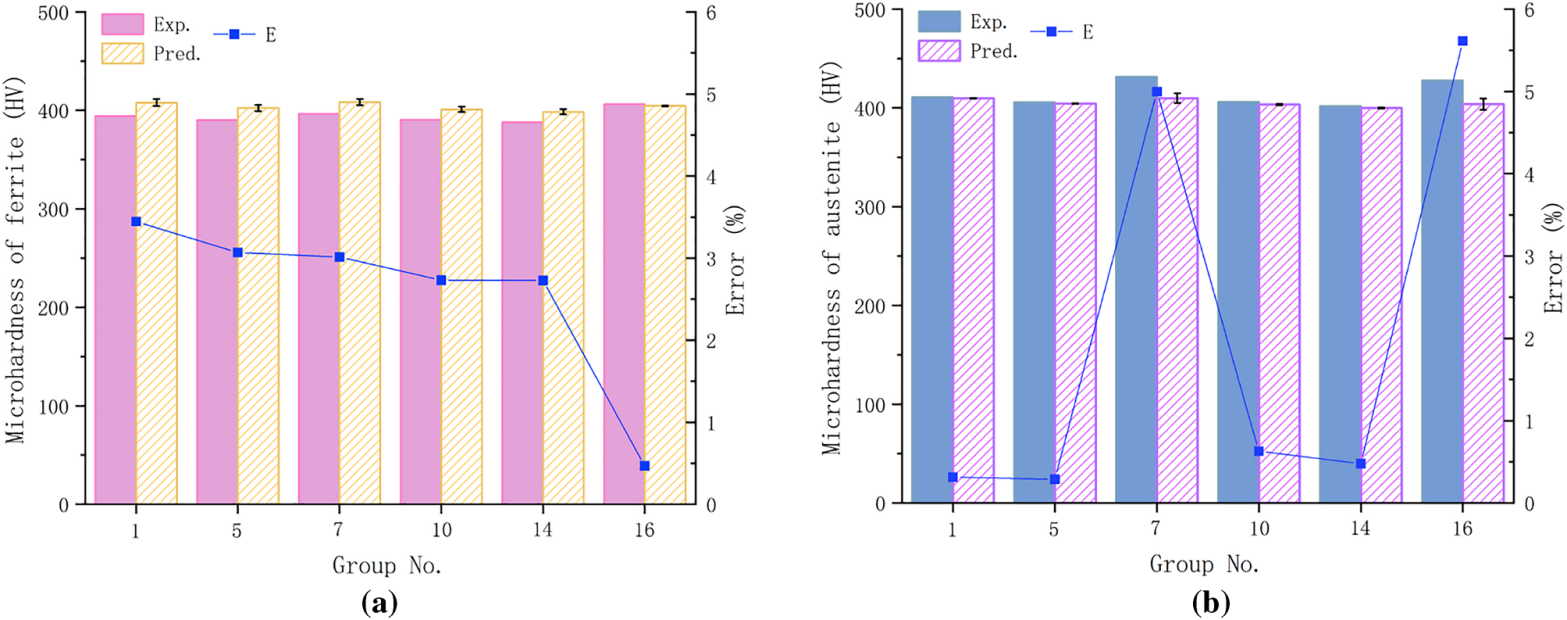
Comparison of model-predicted results with the experimentally measured data: (**a**) Microhardness of ferrite (**b**) Microhardness of austenite.

To characterize and analyze the microhardness of shear bands in the S32760 chip, we collected chip samples from six cutting experiments in groups 1, 5, 7, 10, 14, and 16. After embedding the chips in resin and polishing and etching, we measured the microhardness of the ferrite and austenite in the chip shear band using a Vickers microhardness tester (shown in Fig. 11). Predicted and experimental values on microhardness of the two-phase in the chip shear plane of the six groups are shown in Table 8 and compared in Fig. 12.

**Table 8:** Predicted and experimental values on microhardness of the two-phase in chip shear band.

Experimental group number	1	5	7	10	14	16
Measured microhardness of ferrite /HV	394.27	390.34	396.35	390.45	387.92	406.48
Measured microhardness of austenite /HV	410.98	405.66	431.4	405.97	401.75	427.86
Predicted microhardness of ferrite /HV	407.85	402.32	408.29	401.11	398.50	404.58
Predicted microhardness of austenite /HV	409.69	404.49	409.85	403.42	399.83	403.84
Errors in ferrite microhardness/%	3.4453	3.0687	3.0134	2.7314	2.7269	0.4677
Errors in austenite microhardness/%	0.3144	0.2886	4.9963	0.6285	0.4791	5.6139

In our experimental findings, it was observed that the most significant divergence between the observed and functionally predicted values was evident in the case of austenite, amounting to 5.6%, whereas for ferrite, the difference was 3.4%. It is worth noting that both of these values were below the acceptable error rate of 6%. This discovery has prompted us to assert that our least squares regression equation, founded on cutting dosage, not only exhibits precision but also demonstrates its practicality in predicting the microhardness of both phases within S32760 chips.

### The influence of cutting dosages on the microhardness of two-phase in the chips

**Figure 13:**
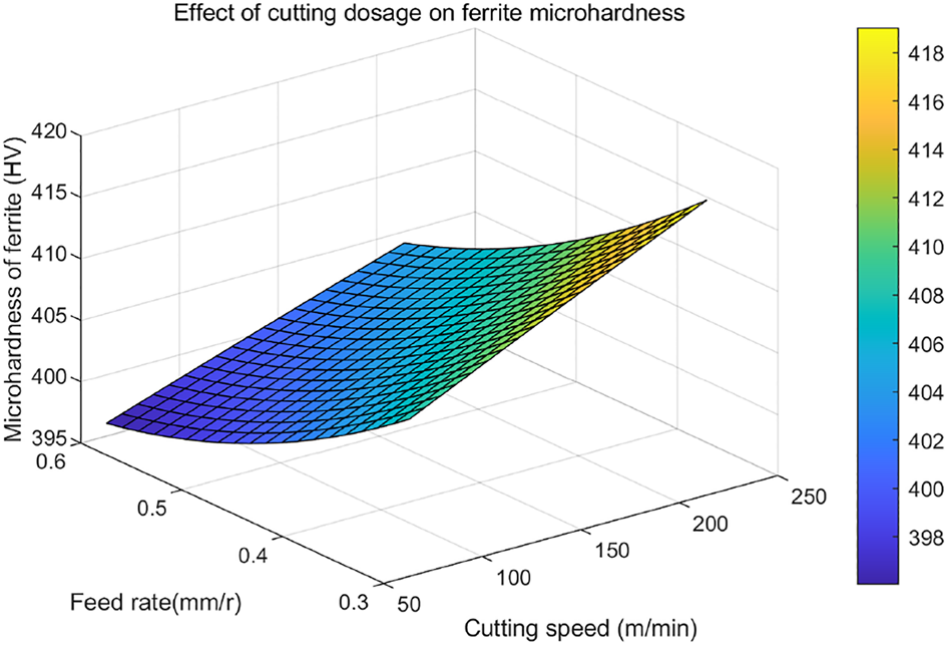
The interactive effect of cutting dosages on the microhardness ($HV_{1}$) of ferrite in the chip shear band.

The interaction between cutting speed ($v_{c}$) and feed ($f$) on the microhardness of ferrite ($HV_{1}$) in the chip shear plane is shown in Fig. 13. As illustrated in the figure, it is evident that the microhardness of ferrite exhibits a discernible increase as cutting speed escalates. With the increase in feed rate, it first decreases and then increases, and the change is relatively slow. Hence, it can be deduced that in comparison to feed rate, cutting speed exerts a more substantial influence on the microhardness of ferrite. In addition, it can be observed that under the same cutting conditions, the maximum microhardness value of ferrite is at the intersection of the minimum feed rate and the maximum cutting speed.

**Figure 14:**
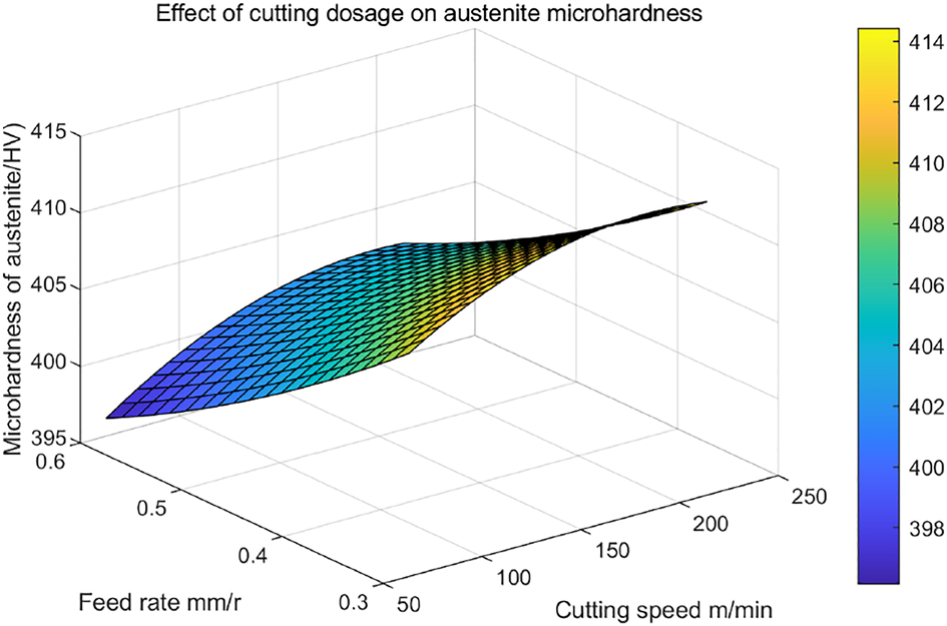
The interactive effect of cutting dosages on the microhardness ($HV_{2}$) of austenite in the chip shear band.

The interaction between cutting speed ($v_{c}$) and feed ($f$) on the microhardness of austenite ($HV_{2}$) at the chip shear plane is shown in Fig. 14. As depicted in the figure, it is evident that the microhardness of austenite initially experiences an increase and subsequently declines with an escalation in cutting speed, with this alteration being more pronounced. As the feed rate increases, there is a continuous decrease in microhardness, with this transformation occurring at a comparatively gradual pace. Hence, it can be inferred that in comparison to the feed rate, cutting speed exerts a more substantial impact on the microhardness of austenite.

According to the third section of this paper, the effect of cutting dosage on the physical field distribution can be observed: when the cutting speed is constant, the increase of feed rate will reduce the strain and strain rate in the shear plane, and the effect of feed rate on temperature is not significant, thus decreasing the microhardness of austenite and ferrite in the shear plane. When the feed rate is constant, the increase in cutting speed will cause an increase in strain and strain rate in the shear plane, leading to an increase in the microhardness of both phases in the shear plane. At the same time, the increase in cutting speed will cause an increase in the average temperature in the shear plane, reducing the microhardness. The changes in the effects of cutting speed and feed rate on the microhardness of ferrite and austenite are different under the same cutting conditions.

## Conclusion

1. We developed a two-phase equipartition shear zone model for orthogonal cutting, using the S32760 two-phase constitutive model as the basis. The shear angle $\varphi$, deformation coefficient $C_{Oxley}$, and the ratio of secondary deformation zone thickness to chip thickness $\delta$ in the shear plane are calculated iteratively using cutting parameters, tool geometry, and material properties. A cutting force prediction model was established based on the iterative calculation results. The average prediction error for the main cutting force $F_{c}$ is 3.4%, and for the cutting force $F_{t}$ is 3.5% when compared to their respective experimental values. The validity of the S32760 orthogonal cutting equidistant shear zone model, which is based on Oxley's theory and accounts for the two-phase properties of materials, has been verified.
2. A mapping relationship between multi-physical fields and cutting parameters was established based on the equipartition shear zone model for orthogonal cutting with two-phase flow stress characteristics. An augmentation in cutting speed leads to a reduction in the shear angle, concomitant with an elevation in the average temperature, shear strain, and shear strain rate of the shear plane. Conversely, increasing the feed rate causes an increase in the shear angle, concomitant with a reduction in shear strain, and shear strain rate, with minimal impact on the average temperature of the shear plane. These observed trends in shear strain, shear strain rate, and average temperature establish a theoretical foundation for predicting and analyzing microhardness in two-phase materials.
3. By establishing a mapping relationship between the two-phase flow stress and hardness prediction model for orthogonal cutting, we obtained the predicted microhardness of the two-phase in the shear plane. Subsequently, using the least square regression method, we established a functional relationship between cutting parameters and the predicted microhardness of the two-phase material. Comparing the experimental and predicted values, we concluded that the prediction model exhibited high accuracy, with a prediction error of less than 6%.
4. This study investigates the variations in microhardness of the two-phase in S32760 under the influence of multiple physical fields during the cutting process. The results indicate that an increase in the feed rate leads to a reduction in shear strain and strain rate within the shear plane, while the impact on temperature is insignificant. Consequently, this leads to a decrease in the microhardness of the two-phase material. On the contrary, an elevation in cutting speed results in elevated shear strain, strain rate, and average temperature within the cutting zone. Thus, cutting speed exerts a dual impact on the microhardness of the two-phase material.

## Data Availability

All data generated or analyzed during this study are included in this manuscript.
